# Case Report: Thymic neuroendocrine tumor with metastasis to the breast causing ectopic Cushing’s syndrome

**DOI:** 10.3389/fonc.2025.1492187

**Published:** 2025-02-25

**Authors:** Aleksandra Zdrojowy-Wełna, Marek Bolanowski, Joanna Syrycka, Aleksandra Jawiarczyk-Przybyłowska, Justyna Kuliczkowska-Płaksej

**Affiliations:** ^1^ Department and Clinic of Endocrinology and Internal Medicine, Wroclaw Medical University, Wroclaw, Poland; ^2^ Affidea PET/CT Diagnostic Center, Wrocław, Poland

**Keywords:** ectopic Cushing`s syndrome, thymic neuroendocrine tumor, thymic NET, ectopic ACTH secretion, case report

## Abstract

Ectopic adrenocorticotropic hormone secretion (EAS) is responsible for approximately 10%–18% of Cushing’s syndrome cases. Thymic neuroendocrine tumors (NETs) comprise 5%–16% of EAS; therefore, they are very rare and the data about this particular tumors is scarce. We present a case of a 34-year-old woman with a rapid onset of severe hypercortisolism in April 2016. After initial treatment with a steroid inhibitor (ketoconazole) and diagnostics including ^68^Ga DOTA-TATE PET/CT, it was shown to be caused by a small thymic NET. After a successful surgery and the resolution of all symptoms, there was a recurrence after 5 years of observation caused by a metastasis to the breast, shown in the ^68^Ga DOTA-TATE PET/CT result and confirmed with a breast biopsy. Treatment with a steroid inhibitor (metyrapone) and tumor resection were again curative. The last disease relapse appeared 7 years after the initial treatment, with severe hypercortisolism treated with osilodrostat. There was a local recurrence in the mediastinum, and a thoracoscopic surgery was performed with good clinical and biochemical effect. The patient remains under careful follow-up. Our case stays in accordance with recent literature data, showing that patients with thymic NETs are younger than previously considered and that the severity of hypercortisolism does not correlate with the tumor size. The symptoms of EAS associated with thymic NET may develop rapidly and may be severe as in our case. Nuclear medicine improves the effectiveness of the tumor search, which is crucial in successful EAS therapy. Our case also underlines the need for lifelong monitoring of patients with thymic NETs and EAS.

## 1 Introduction

Ectopic adrenocorticotropic hormone secretion (EAS) represents between 9% and 18% of adrenocorticotropic hormone (ACTH)-dependent Cushing’s syndrome (CS) cases (1–3). The tumors secreting ACTH may occur in many locations and present with different histopathological differentiation, resulting in various clinical outcomes. In the past, most of the EAS cases were associated with small cell lung cancer, characterized by rapid tumor progression and unfavorable prognosis. Recently, well-differentiated neuroendocrine tumors (NETs) from the foregut prevail in the clinical series of EAS, with most common locations in the lungs, thymus, and pancreas (1).

EAS is often associated with severe hypercortisolism. Typical Cushing’s appearance may not be present due to the rapid onset of the disease. Patients with this type of hypercortisolism need urgent treatment because they have the highest mortality of all forms of CS (4). A retrospective review of 43 patients with EAS reported deaths in 27 patients (62.8%) and a median overall survival of 32.2 months. The leading causes of mortality were the progression of primary malignancies and systemic infections; two patients died from pulmonary embolism (5).

Prompt surgical removal of the tumor secreting ACTH is the mainstay of the therapy. However, finding the tumor causing EAS can be challenging due to its small size and variety of locations. Most authors recommend a combination of computed tomography (CT) scanning of the chest, abdomen, and pelvis, with additional magnetic resonance imaging (MRI) of the pituitary, as the first-line examinations (1, 6, 7). However, the sensitivity of standard imaging modalities is suboptimal (8). In the analysis of 231 patients with EAS, cross-sectional imaging revealed the source of ACTH in 52.4% of them at initial evaluation, and another 29% was found during follow-up or due to nuclear medicine functional imaging, while 18.6% remained occult (9). Nuclear medicine improves the sensitivity of conventional radiology in the case of EAS, with the use of 18-fluorodeoxyglucose (^18^F-FDG) positron emission tomography (PET)/CT (^18^F-FDG PET/CT) expected to be useful in identifying EAS tumors with high proliferative activity and ^68^gallium-labeled somatostatin analogues (^68^Ga DOTA-TATE) PET/CT with the potential to detect NETs. In the head-to-head comparison, the detection rate of the source of EAS was 75% for ^68^Ga DOTA-TATE and 60% for ^18^F-FDG PET/CT, while the highest sensitivity (90%) was achieved when both methods were combined (10).

Thymic NETs comprise 2%–5% of all thymic neoplasms and may cause some paraneoplastic syndromes, with the most frequent being myasthenia gravis, syndrome of inappropriate antidiuretic hormone secretion, and hypercortisolism (11). EAS associated with thymic NETs are rare, representing between 5% and 16% of EAS in published case series (1). Because of the rarity and heterogeneity of the disease, no evidence-based guidelines are available.

We present a case of a patient with thymic NET causing EAS, with metastasis to the breast after 5 years of post-surgical remission and another local recurrence 7 years after the first operation.

Our case is unique because thymic NETs causing EAS are known as an aggressive disease with a median recurrence time of 24 months after thymectomy (12). There are only a few cases described of metastases to the breast from thymic NETs causing EAS (13–16). Moreover, ^68^Ga-SSTR PET/CT was very helpful in detecting both primary and metastatic ectopic ACTH-secreting tumor, which underlines its role in the diagnostic workout of EAS.

## 2 Case description

A 32-year-old woman with no relevant medical history was admitted to the endocrinology department in April 2016 due to the rapid onset of symptoms: weight gain, hypertension, skin changes, and oligomenorrhoea.

The measurements at initial physical examination were as follows: body mass index (BMI)—29 kg/m^2^, blood pressure—180/90 mmHg, and heart rate—88/min. She had plethora, acne, moon face, buffalo hump, central obesity, many red striae in the abdominal area, and mild hirsutism. The baseline laboratory findings are presented in **Table 1**, with hypokalemia, diabetes, leukocytosis, high levels of serum cortisol, ACTH, and chromogranin A, and increased urine-free cortisol (UFC) secretion. There was no suppression of serum cortisol or UFC after a high-dose dexamethasone test. ACTH-dependent CS was diagnosed, and EAS was suspected. The patient’s family history was negative for endocrine diseases or genetic disorders.

**Table 1:** Laboratory results at diagnosis (April 2016).

Parameter	Value	Reference range
Leukocytes (G/L)	22.37	4–10.5
Neutrophiles (%)	82%	60–70
Blood potassium (mmol/L)	2.8	3.8–5
FBG (mg/dL)	97	76–100
Glucose (mg/dL) in 75 g oral glucose tolerance test	210	<140
ACTH (pg/ml)	425	0–46
8:00 PTC (µg/dL)	104.6	10–25
24:00 PTC (µg/dL)	79	<1.8
24-h UFC (µg/24 h)	3088	14–75
PTC after HDDST (nmol/L)	79.4	<1.8
ACTH after HDDST (ng/L)	215	–
UFC after HDDST (µg/24 h)	7271	–
Chromogranin A (µg/L)	437	0–100

ACTH, adrenocorticotrophic hormone; FBG, fasting blood glucose; HDDST, high-dose dexamethasone suppression test; PTC, plasma total cortisol; UFC, urine-free cortisol.

**Figure 1:**
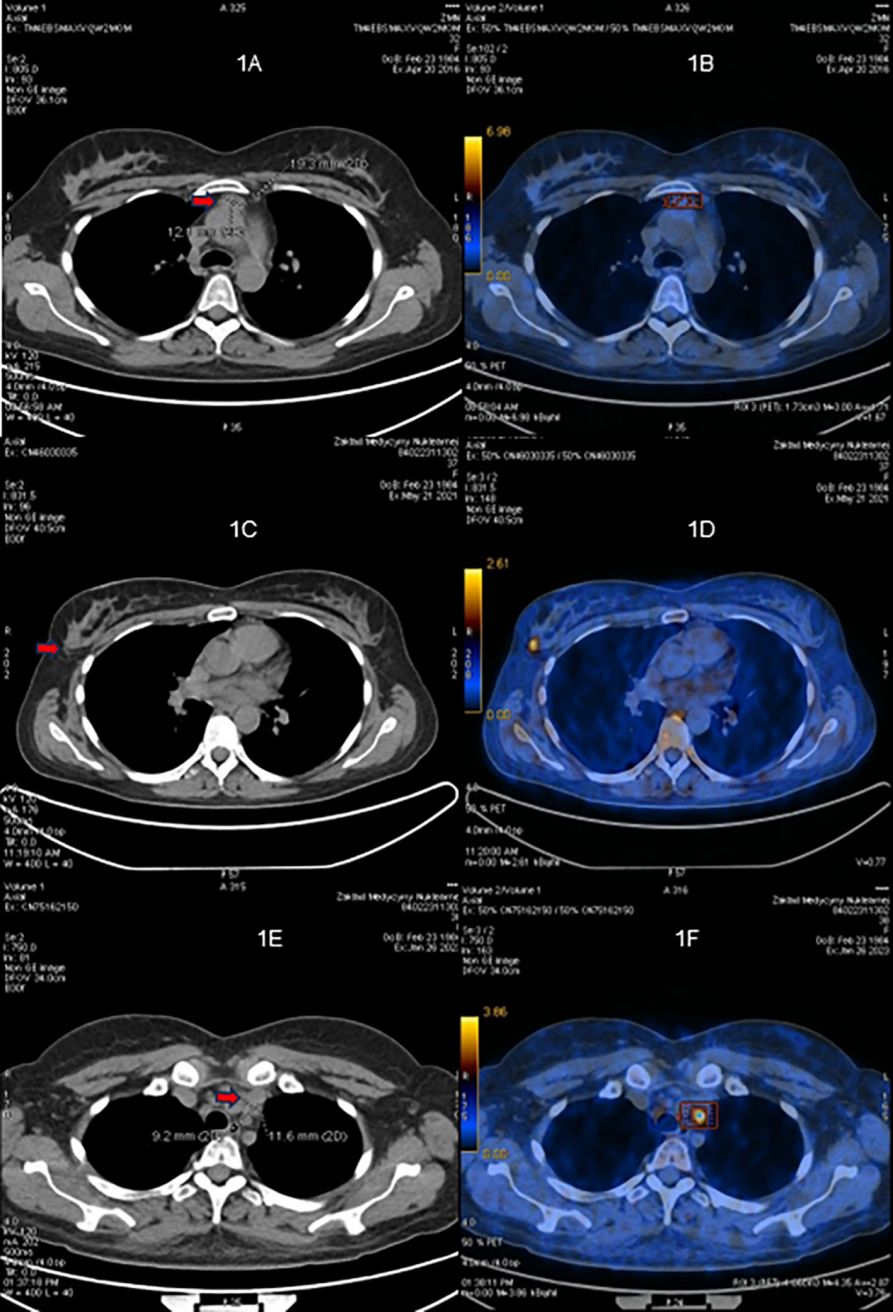
^68^Ga-DOTATATE PET/CT scans. **(A, B)** Before the first surgery (April 2016). **(C, D)** Before the second surgery (May 2021). **(E, F)** Before the third surgery (January 2023).

The first-line cross-sectional imaging studies (chest, abdomen, and pelvis CT and MRI of the pituitary gland) did not reveal the source of ACTH. Only a symmetrical enlargement of adrenals was observed. ^68^Ga DOTA-TATE PET/CT revealed an oval lesion in the anterior mediastinum (1.9 × 1.3 cm) with a subtle overexpression of somatostatin receptors (SUV max. 2.8, **Figures 1A, B**). The chest MRI confirmed a mass 1.5 × 2.0 × 2.5 cm, with high T2-weighted signal and high contrast enhancement, suggestive of NET. The patient was given ketoconazole (600 mg daily), spironolactone, potassium supplementation, antihypertensive drugs, and thromboembolic prophylaxis. In June 2016, thoracoscopic removal of the mediastinal tumor was performed. In the histopathological examination, the tumor was encapsulated, without evidence of invasion, and no lymph node metastases were described. The immunophenotype of the tumor was as follows: CgA (+), Syn (+), CKAE1+E3 (+) “dot-like”, S100 (-), calcitonin (-), EMA (+/-), Ki67 3% to 4% in hot spots, no necrosis, mitotic index 0/10HPF with conclusion: thymic NET—typical carcinoid (low-grade). The presence of paraganglioma was also taken into consideration, as such cases were described (17). However, the significant reaction with cytokeratin and lack of S100 protein expression made this diagnosis less probable.

The postoperative morning serum cortisol concentration was below 5 µg/dL, indicating biochemical remission. The patient received hydrocortisone substitution for a month. The clinical signs of CS disappeared, and there was a normalization of UFC.

During 5 years of follow-up, the patient got pregnant and delivered a healthy child. Genetic counseling was performed, and no germline mutation of MEN1 gene was identified. Other clinical manifestations of MEN1 (like primary hyperparathyroidism and pituitary secreting tumors) were excluded.

In May 2021, the patient experienced a sudden recurrence of CS symptoms. The laboratory findings confirmed severe hypercortisolism (**Table 2**); therefore, treatment with steroid inhibitor metyrapone was administered. The patient tolerated only 750 mg daily; there were side effects (skin rash and tachycardia) with higher doses. The chest MRI revealed no recurrence in the location of the primary tumor, only a lesion in the right breast (1.2 × 1.0 × 1.1 cm) with atypical contrast enhancement. The ^68^Ga-DOTA-TATE PET/CT result showed a subtle overexpression of the tracer (SUV max 1.9) in the right breast (**Figures 1C, D**). Breast ultrasonography confirmed a hypoechogenic, hypervascular mass in the right breast, BIRADS 3/4, diagnosed as NET in the breast biopsy. The tumor was removed in July 2021 without complications. The histopathological samples were compared with the primary lesion, confirming the metastasis from thymic NET to the breast—tumor size 0.7 × 1.5 cm, clear surgical margins (8 mm) with Ki67 3% (NET G2), and no lymph node metastases. After the breast surgery, the cortisol levels normalized in blood and urine and the CS symptoms disappeared. ^18^F-FDG PET/CT and ^68^Ga-DOTA-TATE PET/CT were performed, showing no pathological increase of radiotracer uptake in post-operative locations or mediastinal lymph nodes. The patient consulted with the oncology team, and no adjuvant therapy was recommended.

**Table 2:** Laboratory results during 7 years of observation.

	April 2016	October 2016	May 2021	October 2021	January 2023	February 2023	September 2023
24:00 PTC (µg/dL)	91	0.7	107	1.3	60	2.1	2.5
24-h UFC (µg/24 h)	3,088	63	27,475	28	15,003	33	30
ACTH (pg/ml)	425	8	479	33	489	30	25
Chromogranin A (µg/L)	437	42	>900	8	>900	165	38

ACTH, adrenocorticotrophic hormone; PTC, plasma total cortisol; UFC, urine-free cortisol.

The next recurrence of the disease occurred in February 2023, with the symptoms developing suddenly during a very short period (1 to 2 weeks), additionally with significant mental deterioration (concentration disorders, anxiety, severe mood swing). The laboratory findings confirmed excessive hypercortisolism (**Table 2**). The patient was given osilodrostat (the initial dose was 20 mg daily but later reduced to 10 mg daily for 2 weeks until surgery) and symptomatic treatment with good clinical and biochemical effect. The ^68^Ga-DOTA-TATE PET/CT result showed a slightly increased uptake of the tracer in the left mediastinum, between cervical vessels, 0.9 × 1.2 cm (**Figures 1E, F**)—probably a local recurrence. Thoracotomy was performed in February 2023, with subsequent clinical and biochemical improvement (**Table 2**). In the histopathological examination, mediastinal NET G1 was diagnosed, without necrosis, mitotic activity 0/2 mm^2^, immunophenotype CgA (+), CD56 (+), Ki 67 1%, CK AE1/AE3 (+), CD117 (+), p40 (-), TdT (-), PAX8 (-), and the presence of tumor cell embolism in the vessels. One metastatic lesion was found in the pericardium (the maximal dimension of the tissue was 13 mm, resected radically). Two metastatic lesions in the fat tissue were found (one tissue fragment from the mediastinum, max. 16 mm diameter, and the second tissue fragment was surrounding the jugular vein, max. diameter up to 40 mm, both resected radically). Two of the 10 resected lymph nodes had metastatic lesions: one from the area of the jugular vein, diameter 11 mm, with capsular invasion, and the second lymph node N2R with capsular invasion, both resected radically. The symptoms of hypercortisolism disappeared, and the cortisol values were normalized after the operation. The patient is currently under careful monitoring, without signs of clinical or biochemical recurrence. ^68^Ga-DOTA-TATE PET/CT is performed every 6 months.

## 3 Discussion

Our case is representative for thymic NETs causing EAS presented in literature, but it also shows some distinct features, giving new insight into this rare condition.

In recent series, ACTH-secreting thymic NETs occurred often in young adults, like our patient. The typical age of presentation is 21–35 years in the largest case series, and 7.4% were children under 15 years (12, 13). In contrast, the former series of thymic NETs showed a peak incidence in the sixth decade of life (11).

ACTH-secreting thymic NETs show a slight male preponderance (58.6%); however, the patient’s gender does not seem to relate with the disease outcome (12). There was only an association between male sex and larger tumor size preoperatively as found in one case series (13).

Thymic NETs causing EAS are very rarely associated with MEN1; we have also excluded it in our patient. On the contrary, 30% of thymic NETs not associated with CS are found in patients with MEN1, mostly male smokers (18). It is not clear why thymic NETs with EAS are less likely caused by MEN1 gene mutation, but the possibility of this genetic predisposition should always be taken into consideration.

Thymic NETs associated with EAS are generally considered aggressive, presenting significant cellular atypia in the histopathological examination (19). However, the biology of the tumors is variable. In the histopathological examination of 92 thymic NETs secreting ACTH, the most common subtype was atypical NET (46.7%), while 30.4% of the cases were typical NETs and 21.7% were carcinomas, with the median Ki-67 10%, ranging from 1% to 40%. The median tumor size among 112 patients was 4.7 cm, ranging from 1 to 20 cm, and 55.7% of patients had metastases at presentation (12). It proves the significant heterogeneity of the disease.

Our patient had typical NET with small dimensions and localized disease at the time of diagnosis. Despite this, we observed aggressive Cushing’s syndrome with a short duration of symptoms and life-threatening hypokalemia. It has been observed that there is no correlation between tumor size and hormone levels (12). Thymic NETs associated with EAS are often large, which simplifies the diagnosis and localization. However, in the case of incidental sellar mass or small thymic tumor, the differential diagnosis might be difficult. The highest sensitivity in distinguishing thymic EAS from Cushing’s disease was documented in inferior petrosal sinus sampling and corticotropin-releasing hormone (CRH) stimulation test (12, 20).

In severe cases, when small ACTH-secreting NET needs to be found urgently, PET/CT is a very helpful diagnostic tool. In a prospective study comprising 20 patients with histologically proven EAS, the ^68^Ga-DOTATATE PET/CT result correctly identified the tumor in 75%, with SUV max. ranging from 1.4 to 20.7, while the ^18^F-FDG PET/CT findings had a slightly worse result (identified 60% tumors), with SUV max. ranging from 1.8 to 10.0. Those methods are believed to be complementary in case of localization and discrimination of EAS. The ^68^Ga-DOTATATE PET/CT result revealed tumor in six cases with a negative ^18^F-FDG PET/CT result, while the ^18^F-FDG PET/CT procedure was diagnostic in three cases with a negative ^68^Ga-DOTATATE uptake; the combined sensitivity of both methods was 90% (10). The typical first-line diagnostic modalities’ (CT and MRI) sensitivities range from 52% to 66% (9). Our case remains in accordance with those results, showing difficulties in localizing the ACTH source in first-line radiological methods and with ^68^Ga-DOTATATE PET/CT being the most useful diagnostic tool. It should also be noted that the ^68^Ga-DOTATATE uptake was only mildly elevated both in primary tumor and its recurrences despite excessive hormonal activity. We did not perform ^18^F-FDG PET/CT until second operation, as it was believed to be rather helpful in poorly differentiated tumors and ^68^Ga-DOTATATE PET/CT was diagnostic. Later, we performed it in search for other metastatic tumors, but the examination showed no tumor spread.

The recommended treatment of thymic NETs regarded radically resectable is thymectomy by median sternotomy or thoracotomy and lymph node dissection (11, 21, 22). According to the last version of the ESMO Guidelines, available literature suggests no benefit from adjuvant therapy in ThCs. The majority of the authors of the Guidelines panel suggest individually discussing eventual postoperative therapies, including RT and/or systemic therapies, balancing the pros and cons only in selected patients with advanced stage R0 or R1-2 resection (22). Data on systemic therapies in thymic NETs are scarce; therefore, they should be discussed in a multidisciplinary expert team in case of morphologically progressive tumors, high tumor burden, or refractory hormonal syndromes. Somatostatin analogs are recommended as the first-line systemic therapy in typical carcinoids (22). We considered the adjuvant therapy with somatostatin analogs; however, due to the low uptake in PET examination and complete resolution of symptoms as well as the radical type of surgical removal, we did not decide to initiate such therapy. Other systemic treatment options include everolimus (second line in typical carcinoids or first line in atypical carcinoids), chemotherapy, peptide receptor radionuclide therapy (PRRT), and interferon-α (22, 23). There is also data on the benefits of combining long-acting lanreotide with temozolomide in progressive thymic NETs (24).

Due to the variable availability of steroid inhibitors during the course of the disease, our patient received three different preparations at each disease relapse. Both ketoconazole and osilodrostat were well tolerated and reduced the hypercortisolism within a few days, but metyrapone caused significant side effects (see below—”Patient’s perspective”), and it was not possible to normalize the cortisol values with this steroid inhibitor. It is worth noting that when using the most recent steroid inhibitor—osilodrostat—we initiated the therapy with a high dose without a previous dose titration. This strategy might be used in the case of severe hypercortisolism and proved effective and safe in our patient (25).

Most commonly, metastases from thymic NET producing ACTH are localized in lymph nodes, bone, lung, pleura, and, less commonly, liver and parotid gland (13). There are very few cases of EAS-related thymic NETs with breast metastases described in the literature, with some histopathological variability (one case related to atypical carcinoid, another to combined large-cell neuroendocrine carcinoma and atypical carcinoid, and third case of neuroendocrine carcinoma). All of them were female patients between 24 and 36 years of age, with mediastinal lymph nodes metastases at the time of presentation; one also had distant metastases to the bones (13–15). Contrary to the reported cases, our patient had typical carcinoid (confirmed by three independent pathologists from different centers) but similarly presented with severe hypercortisolism. It suggests that there is no connection between tumor differentiation and the severity of hypercortisolism. Interestingly, in a review of 661 patients with metastatic NETs from Sweden, there were 20 patients with NETs and breast metastases, and among them only one case of thymic NET (Ki 67 12%), but without EAS. A total of 11 patients with breast metastases had a primary tumor in the small intestine and eight in the lung (16).

Our case underlines the necessity of long-term follow-up in EAS, as the recurrences occurred 5 and 7 years after the initial successful treatment. According to guidelines, follow-up after treatment of thymic NETs should be life-long (22).

The strength of our report is the presentation of a thymic NET with metastasis to the breast, diagnosed and treated with many currently available tools and with a long period of follow-up. The limitation is the low number of other similar cases to compare, which is a consequence of the rarity of this disease.

In conclusion, our case proves that thymic NETs with EAS might present in young patients with well-differentiated character in histopathological examination and severe, life-threatening hypercortisolism despite the small size of the primary lesion. ^68^Ga-DOTATATE PET/CT is a very helpful tool to localize the tumor. Finally, life-long follow-up should be performed despite complete remission after surgery.

## 4 Patient’s perspective

The first symptoms that I observed were face edema and mood changes. I rapidly lost muscle mass (approximately 6 kg in 2 weeks), and I was not able to climb stairs, especially with my child’s pram. The most difficult to accept were changes in my appearances—hirsutism, losing hair, changes of my facial features. My sense of pain (for example, during medical procedures) was diminished. Other disruptive symptoms were intensive sweating, increased appetite, thirst, brain fog, and digestive problems. At every relapse, the disease manifestations were fluctuating, all of them intensifying at the same time, which was very difficult for me. Also stress evoked disease symptoms. I experienced a strange feeling of warm during cortisol outbursts.

As for the treatment, I did not tolerate metyrapone well. I had skin rash, anxiety attacks with heart palpitations, and a metallic taste in my mouth. Other drugs (ketoconazole, osilodrostat) were better for me.

After operations of the relapses, the symptoms diminished very quickly, especially the most difficult ones. My blood pressure and glycemia normalized within a few days. Other manifestations, like loss of hair or skin changes, persisted up to 3 months.

## Data Availability

The datasets presented in this article are not readily available because the data are potentially identifiable. Requests to access the datasets should be directed to Aleksandra Zdrojowy-Wełna, aleksandra.zdrojowy-welna@umw.edu.pl.

## References

[1] Young J Haissaguerre M Viera-Pinto O Chabre O Baudin E Tabarin A. Management of endocrine disease: Cushing’s syndrome due to ectopic ACTH secretion: an expert operational opinion. Eur J Endocrinol. (2020) 182:29–58. doi: 10.1530/EJE-19-0877 31999619

[2] Feelders R Sharma S Nieman L. Cushing`s syndrome: epidemiology and developments in disease management. Clin Epidemiol. (2015) 7:281–93. doi: 10.2147/CLEP.S44336 PMC4407747 25945066

[3] Lacroix A Feelders RA Stratakis CA Nieman LK. Cushing’s syndrome. Lancet Lond Engl. (2015) 386:913–27. doi: 10.1016/S0140-6736(14)61375-1 26004339

[4] Javanmard P Duan D Geer EB. Mortality in patients with endogenous cushing’s syndrome. Endocrinol Metab Clin North Am. (2018) 47:313–33. doi: 10.1016/j.ecl.2018.02.005 29754634

[5] Ejaz S Vassilopoulou-Sellin R Busaidy NL Hu MI Waguespack SG Jimenez C. Cushing syndrome secondary to ectopic adrenocorticotropic hormone secretion: the University of Texas MD Anderson Cancer Center Experience. Cancer. (2011) 117:4381–9. doi: 10.1002/cncr.v117.19 PMC3134535 21412758

[6] Sookur PA Sahdev A Rockall AG Isidori AM Monson JP Grossman AB. Imaging in covert ectopic ACTH secretion: a CT pictorial review. Eur Radiol. (2009) 19:1069–78. doi: 10.1007/s00330-008-1274-5 19137302

[7] Yogi-Morren D Habra MA Faiman C Bena J Hatipoglu B Kennedy L. Pituitary MRI findings in patients with pituitary and ectopic ACTH-dependent cushing syndrome: does A 6- mm pituitary tumor size cut-off value exclude ectopic acth syndrome? Endocr Pract. (2015) 21:1098–103. doi: 10.4158/EP15662.OR 26121435

[8] Ilias I Torpy DJ Pacak K Mullen N Wesley RA Nieman LK. Cushing’s syndrome due to ectopic corticotropin secretion: twenty years’ Experience at the national institutes of health. J Clin Endocrinol Metab. (2005) 90:4955–62. doi: 10.1210/jc.2004-2527 15914534

[9] Isidori AM Sbardella E Zatelli MC Boschetti M Vitale G Colao A. Conventional and nuclear medicine imaging in ectopic cushing’s syndrome: A systematic review. J Clin Endocrinol Metab. (2015) 100:3231–44. doi: 10.1210/JC.2015-1589 PMC4570166 26158607

[10] Liu Q Zang J Yang Y Ling Q Wu H Wang P. Head-to-head comparison of 68Ga- DOTATATE PET/CT and 18F-FDG PET/CT in localizing tumors with ectopic adrenocorticotropic hormone secretion: a prospective study. Eur J Nucl Med Mol Imaging. (2021) 48:4386–95. doi: 10.1007/s00259-021-05370-8 34146130

[11] Gaur P Leary C Yao JC. Thymic neuroendocrine tumors: a SEER database analysis of 160 patients. Ann Surg. (2010) 251:1117–21. doi: 10.1097/SLA.0b013e3181dd4ec4 20485130

[12] Guerrero-Pérez F Peiró I Marengo AP Teulé A Ruffinelli JC Llatjos R. Ectopic Cushing’s syndrome due to thymic neuroendocrine tumours: a systematic review. Rev Endocr Metab Disord. (2021) 22:1041–56. doi: 10.1007/s11154-021-09660-2 33961211

[13] Neary NM Lopez-Chavez A Abel BS Boyce AM Schaub N Kwong K. Neuroendocrine ACTH-producing tumor of the thymus—Experience with 12 patients over 25 years. J Clin Endocrinol Metab. (2012) 97:2223–30. doi: 10.1210/jc.2011-3355 PMC3387392 22508705

[14] Dzialach L Wojciechowska-Luzniak A Maksymowicz M Witek P. Case report: A challenging case of severe Cushing’s syndrome in the course of metastatic thymic neuroendocrine carcinoma with a synchronous adrenal tumor. Front Endocrinol. (2024). doi: 10.3389/fendo.2024.1399930/full PMC11211248 38948516

[15] Gaur S Ayyappan AP Nahleh Z. Breast metastases from an adrenocorticotropic hormone secreting thymic neuro-endocrine tumor. Breast Dis. (2013) 34:81–6. doi: 10.3233/BD-130354 23948804

[16] Crona J Granberg D Norlén O Wärnberg F Stålberg P Hellman P. Metastases from neuroendocrine tumors to the breast are more common than previously thought. A diagnostic pitfall? World J Surg. (2013) 37:1701–6. doi: 10.1007/s00268-013-2037-2 23592057

[17] Li B Yan Z Huang H. Case report: an unusual case of ectopic ACTH syndrome caused by mediastinal paraganglioma. Front Endocrinol. (2021) 12:790975. doi: 10.3389/fendo.2021.790975 PMC8769203 35069444

[18] Ferolla P Falchetti A Filosso P Tomassetti P Tamburrano G Avenia N. Thymic neuroendocrine carcinoma (carcinoid) in multiple endocrine neoplasia type 1 syndrome: the Italian series. J Clin Endocrinol Metab. (2005) 90:2603–9. doi: 10.1210/jc.2004-1155 15713725

[19] Moran CA Suster S. Neuroendocrine carcinomas (Carcinoid tumor) of the thymus. Am J Clin Pathol. (2000) 114:100–10. doi: 10.1309/3PDN-PMT5-EQTM-H0CD 10884805

[20] Kakade HR Kasaliwal R Jagtap VS Bukan A Budyal SR Khare S. Ectopic acth- secreting syndrome: A single-center experience. Endocr Pract. (2013) 19:1007–14. doi: 10.4158/EP13171.OR 24013993

[21] Girard N. Neuroendocrine tumors of the thymus: the oncologist point of view. J Thorac Dis. (2017) 9:1491–500. doi: 10.21037/jtd.2017.08.18 PMC5690949 29201452

[22] Baudin E Caplin M Garcia-Carbonero R Fazio N Ferolla P Filosso PL. Lung and thymic carcinoids: ESMO Clinical Practice Guidelines for diagnosis, treatment and follow-up. Ann Oncol Off J Eur Soc Med Oncol. (2021) 32:439–51. doi: 10.1016/j.annonc.2021.01.003 33482246

[23] Ferolla P Brizzi MP Meyer T Mansoor W Mazieres J Do Cao C. Efficacy and safety of long-acting pasireotide or everolimus alone or in combination in patients with advanced carcinoids of the lung and thymus (LUNA): an open-label, multicentre, randomised, phase 2 trial. Lancet Oncol. (2017) 18:1652–64. doi: 10.1016/S1470-2045(17)30681-2 29074099

[24] Ferolla P Berruti A Spada F Brizzi MP Ibrahim T Marconcini R. Efficacy and safety of lanreotide autogel and temozolomide combination therapy in progressive thoracic neuroendocrine tumors (Carcinoid): results from the phase 2 ATLANT study. Neuroendocrinology. (2023) 113:332–42. doi: 10.1159/000526811 36044870

[25] Fleseriu M Biller BMK. Treatment of Cushing’s syndrome with osilodrostat: practical applications of recent studies with case examples. Pituitary. (2022) 25:795–809. doi: 10.1007/s11102-022-01268-2 36002784 PMC9401199

